# The Allergen Bureau approach to allergen cross contact risk assessment – our journey

**DOI:** 10.1186/2045-7022-1-S1-O2

**Published:** 2011-08-12

**Authors:** Kristen Grinter, Fiona Fleming, Robin Sherlock, Neil Smith

**Affiliations:** 1 Allergen Bureau, Allergen Bureau Chairperson, Sydney, Australia; 2 Allergen Bureau, Management Committee, Sydney, Australia; 3 Allergen Bureau, Management Committee, Brisbane, Australia; 4 Allergen Bureau, Management Committee, Melbourne, Australia

## 

The Allergen Bureau was established in 2005 and is funded by membership from the Australian and New Zealand food industry. The industry developed Guide to Allergen Management and Labelling is a resource to ensure consistency in manufacturing practices and labelling. VITAL (Voluntary Incidental Trace Allergen Labelling) is a risk tool that was developed against a regulatory background where the labelling of incidental trace cross contact allergens is not mandated. VITAL is a risk based methodology to be used in assessing the impact of allergen cross contact and stipulates a consistent precautionary allergen labelling statement. Application of the VITAL system aims to avoid indiscriminate use of precautionary labelling and contribute to the minimisation of risk to allergic consumers.

VITAL uses an action level grid to assist in determining if the presence of residual protein from allergenic substances through cross contact requires precautionary labelling. VITAL is based on the premise that some products may have foreseeable levels of an allergen present through incidental cross contact, and this will not be labelled where the level is below a specified action level. Due to the wide use and international interest in VITAL, it is important that VITAL be reviewed regularly to ensure that it can be considered to be the best available tool for cross contact allergen risk management.

The purpose of this presentation is to provide information on the status of VITAL today and to outline the review process currently underway .A significant element in this process is the establishment of a scientific expert panel to review current clinical data, population data, and to consider the application of probabilistic modelling to the current action levels. This expert panel will meet in Australia in January 2011 and we hope to be able to report on the outcomes of the first meeting and the next steps in the journey .A consistent approach to cross contact risk assessment is key to ensuring reliability of labelling and consumer confidence and safety.

